# Is postoperative cognitive dysfunction a disease of microglial inflammatory memory? A state-transition model from metabolic stress to epigenetic lock-in

**DOI:** 10.3389/fnmol.2025.1648161

**Published:** 2025-08-06

**Authors:** Xiangyu Wu, Tingye He, Fei He, Li Liu

**Affiliations:** ^1^Department of Anesthesiology, The Affiliated Hospital, Southwest Medical University, Luzhou, Sichuan, China; ^2^Anesthesiology and Critical Care Medicine Key Laboratory of Luzhou, Southwest Medical University, Luzhou, Sichuan, China

**Keywords:** microglial inflammatory memory, epigenetic reprogramming, mitochondrial dysfunction, postoperative cognitive dysfunction, neuroimmune plasticity

## Abstract

Postoperative cognitive dysfunction (POCD) remains a significant challenge in perioperative medicine, especially among older adults. Despite its prevalence, existing models centered on transient neuroinflammation fail to explain why cognitive deficits often persist long after systemic immune responses resolve. This review proposes a new framework: POCD is driven not by ongoing inflammation, but by a stable shift in microglial identity. We describe a closed-loop “inflammatory memory circuit” in which mitochondrial dysfunction, chromatin remodeling, and persistent polarization co-evolve to lock microglia into a hypersensitive, neurotoxic state. Recent studies suggest that surgical trauma triggers mitochondrial damage and mtDNA release, initiating innate immune activation via the cGAS–STING and NLRP3 pathways. These events engage epigenetic machinery—including HDAC3, DNMT3a, and long non-coding RNAs like MEG3—which reinforce transcriptional programs that lower activation thresholds and amplify cytokine output. Sustained M1-like polarization further propagates this loop, driving neuronal injury even in the absence of continued systemic cues. We outline experimental strategies to validate this model, including time-resolved single-cell transcriptomics and chromatin accessibility profiling. Therapeutically, we highlight HDAC inhibitors, SIRT1 agonists, and lncRNA-targeted interventions as potential strategies to disrupt the circuit before state-locking occurs. By reframing POCD as a glial fate transition rather than a transient immune reaction, this model offers mechanistic clarity and opens a path toward time-sensitive, precision interventions.

## 1 Introduction: rethinking POCD through the lens of inflammatory memory

Postoperative cognitive dysfunction continues to pose a significant neurocognitive challenge in the aging surgical population. Despite improvements in anesthetic safety and perioperative care, many patients—particularly the elderly—experience a constellation of cognitive disturbances, including episodic memory lapses, executive dysfunction, and impaired attention that may persist for weeks or even months following otherwise uneventful surgeries (Park et al., 2024; Zeng et al., 2023). Recent high-quality studies estimate that early POCD occurs in approximately 20%–40% of elderly patients after non-cardiac surgery (Miller et al., 2018; Punjasawadwong et al., 2018). Alarmingly, POCD is increasingly recognized as a risk factor for premature mortality and accelerated neurodegenerative trajectories, yet these lasting effects often follow a brief and seemingly reversible physiological insult—transient exposure to anesthesia and surgical trauma (Steinmetz et al., 2009; Yang X. et al., 2022; Zhao et al., 2024).

Traditional mechanistic accounts have focused on acute neuroinflammatory responses, transient blood–brain barrier disruption, and peripheral immune activation. While these mechanisms are undoubtedly involved in the perioperative window, they typically resolve within days (Alam et al., 2018; Zhang Z. et al., 2024). This temporal mismatch between a short-lived systemic insult and a protracted period of cognitive dysfunction suggests the involvement of a more persistent, CNS-intrinsic process (Tan et al., 2021; Wang T. et al., 2025). Indeed, several studies have reported the normalization of peripheral inflammatory markers despite ongoing neurocognitive symptoms, raising the possibility that the insult has induced a more enduring transformation within the central nervous system—particularly in its immune-competent glial population (Femenía et al., 2018; Zhang and Yin, 2023).

This review proposes a conceptual shift: rather than interpreting POCD as a failure of inflammation to resolve, we argue that it reflects a maladaptive form of neuroimmune memory. We hypothesize that microglia, upon exposure to perioperative stress, enter a primed state characterized by chromatin imprinting, altered mitochondrial homeostasis, and a persistent bias toward pro-inflammatory functional states (Huang et al., 2023; Martins-Ferreira et al., 2021; Neher and Cunningham, 2019). This locked inflammatory configuration is not merely a residual of injury but represents an epigenetically stabilized phenotype that continues to influence neural circuits long after the primary trigger has receded (Wu et al., 2023). Mounting evidence supports this notion. Studies in aging and neurodegeneration have shown that microglia retain molecular signatures of past insults, including persistent shifts in mitochondrial reactive oxygen species (mtROS) handling, histone acetylation patterns, and non-coding RNA expression (Cheray and Joseph, 2018; Haley et al., 2019; Nakanishi et al., 2011). These features not only render microglia more reactive to secondary stimuli but constitute an epigenetically maintained “neuroimmune memory” state analogous to trained immunity in peripheral macrophages (Zhang X. et al., 2022). In the context of POCD, this memory may manifest as a sustained distortion of the glial environment—one that perpetuates synaptic dysfunction and impairs recovery, even in the absence of ongoing inflammation (Jung et al., 2023; Qi et al., 2023; Tan et al., 2024).

To better articulate this concept, we introduce the “three-axis inflammatory memory model” which describes a recursive interaction between mitochondrial stress, epigenetic remodeling, and microglial functional state transition. In this framework, mitochondrial perturbation serves as the initial trigger, epigenetic reprogramming functions as the stabilizing layer, and pro-inflammatory microglial behavior acts as the amplifying output (Suo et al., 2025; Ying et al., 2024; Zhang L. et al., 2025). These three axes collectively sustain a pathological feedback loop that resists spontaneous resolution and provides a mechanistic basis for the persistent neurocognitive sequelae observed in POCD. We further propose that this model offers multiple experimentally testable avenues. With the aid of single-cell RNA sequencing, ATAC-seq, and histone modification profiling at distinct postoperative intervals, it becomes possible to chart the epigenetic trajectory of microglial states from injury through recovery (Suo et al., 2025). Additionally, we explore potential therapeutic entry points—including SIRT1–PGC-1α modulators, histone deacetylase inhibitors, and long non-coding RNA silencers—that may help dismantle this locked phenotype (Chen et al., 2020; Yang X. et al., 2021; Ye et al., 2023). Finally, we highlight promising candidate biomarkers—such as circulating mtDNA, histone deacetylase (HDAC) activity levels, and MEG3 expression—that could be leveraged to predict susceptibility and inform personalized intervention strategies (Chen et al., 2020; Ye et al., 2023; Zhang L. et al., 2025).

By reframing POCD as a disease of maladaptive neuroimmune imprinting rather than transient inflammation, we aim to redirect mechanistic inquiry and therapeutic innovation toward the underlying cellular programs that sustain cognitive vulnerability. This shift in perspective may yield not only new scientific insights but also practical approaches to identifying and reversing the memory-encoded pathologies at the core of postoperative brain dysfunction.

## 2 Postoperative inflammation inertia: decoupling stimulus and persistence

### 2.1 Persistence of microglial activation beyond cytokine resolution in preclinical models

Rodent models of surgery-induced neuroinflammation consistently demonstrate that microglial activation persists well beyond the transient wave of peripheral cytokines typically triggered by surgical stress. For instance, studies employing tibial fracture or laparotomy under general anesthesia reveal sustained morphological changes in hippocampal and cortical microglia, accompanied by upregulation of Iba1, CD68, and MHC-II expression for up to 7 days postoperatively (Feng et al., 2017; Qiu et al., 2016; Zhu et al., 2016). By contrast, levels of IL-6, TNF-α, and HMGB1 in serum and cerebrospinal fluid return to baseline within 48–72 h, indicating that the acute inflammatory surge is both sharp and short-lived (Zou et al., 2025). This discrepancy suggests that microglia do not simply mirror the temporal profile of systemic inflammation, but instead undergo a more durable shift in functional state (Qiu et al., 2016). Importantly, behavioral studies in the same models show memory deficits and reduced fear conditioning performance that align temporally with glial activation rather than with cytokine elevation (Meng et al., 2019). These findings challenge linear models of inflammation resolution and raise the possibility that microglia, once activated, may adopt a new operational identity that perpetuates dysfunction in the absence of continued external provocation.

Several candidate mechanisms have been proposed. Mitochondrial damage–associated molecular patterns (mtDAMPs), including extracellular mtDNA and cardiolipin, may persist long after tissue healing and act as latent triggers of microglial reactivity (Lin et al., 2022; Liu Y. et al., 2024). In parallel, metabolic reprogramming—marked by enhanced glycolysis and impaired oxidative phosphorylation—resembles a Warburg-like shift, stabilizing microglia in an energetically pro-inflammatory state (Baik et al., 2019; Yang S. et al., 2021). Transcriptomic analyses further support this view, revealing sustained upregulation of chromatin remodelers and pattern recognition receptors, suggesting that once microglia cross an activation threshold, they remain in a functionally reprogrammed state uncoupled from external stimuli (Miao et al., 2023).

Such evidence supports a paradigm in which microglial activation becomes intrinsically sustained—not by persistent signaling, but by a transition in cellular identity. This “phenotypic inertia” likely reflects chromatin imprinting and metabolic stabilization, establishing a new homeostatic set point for microglial behavior. In this light, the neuroimmune system may serve not merely as a reactive interface, but as a durable memory substrate for prior inflammatory episodes.

### 2.2 Human evidence for central inflammatory inertia following surgery

Although direct human brain sampling in the postoperative context is constrained by ethical and technical limitations, converging lines of evidence suggest that central inflammation may outlast systemic recovery. Positron emission tomography (PET) imaging using radiolabeled translocator protein (TSPO) ligandshas been used in studies in autoimmune encephalitis, neurodegeneration, and tuberous sclerosis complex have revealed prolonged central microglial activation that correlates with cognitive decline, even after peripheral inflammatory markers normalize (Kagitani-Shimono et al., 2023; Rossano et al., 2024; Wang J. et al., 2025). These elevations occur independently of systemic cytokine normalization, pointing to a central mechanism that continues to evolve after peripheral markers subside.

In rodent models simulating clinical surgery, sustained elevations of IL-1β and IFN-γ have been detected in the hippocampus up to 7 days postoperatively, accompanied by microglial accumulation in both the hippocampus and prefrontal cortex (Qiu et al., 2016). Moreover, transcriptomic analysis of hippocampal tissue following IFN-γ-induced microglial priming reveals sustained upregulation of chromatin remodeling enzymes, mitochondrial stress-response genes, and MHC-II antigen presentation pathways, reflecting a persistently activated microglial transcriptional state (Zhang J. et al., 2020).

We propose the term inflammatory inertia to describe this phenomenon: a sustained, stimulus-decoupled microglial activation state that represents a qualitative shift rather than a quantitative extension of inflammation. This is not simply prolonged inflammation in the classical sense; rather, it is a form of neuroimmune state-locking, akin to epigenetic reprogramming in cancer or immune cell exhaustion in chronic viral infections. The inflammatory burden may dissipate, but the cell’s functional identity does not revert (Huang et al., 2023; Neher and Cunningham, 2019; Zhang X. et al., 2022). This perspective alters both our understanding and our therapeutic priorities. The critical determinant of POCD may not be the peak intensity or duration of inflammation, but the point at which microglia cross a regulatory threshold and enter a stable, maladaptive phenotype. Unlike acute inflammation, which is inherently self-limiting, this inertia resists resolution and perpetuates subtle but persistent circuit dysfunction (Jung et al., 2023). In recognizing this phenomenon as a neuroimmune state transition, we shift the therapeutic focus from suppressing cytokine production to intercepting—or reversing—the cellular reprogramming that maintains microglial pathogenicity. This reorientation also informs biomarker discovery: rather than tracking inflammatory surges, future diagnostics should aim to detect epigenetic or metabolic indicators of glial state locking.

Taken together, existing preclinical and mechanistically relevant clinical findings support a revised model of postoperative neuroinflammation—not as a fading echo of peripheral insult, but as a form of innate immune memory embedded within the brain’s microglial population. This framework reflects a shift from stimulus–response coupling to a persistent, self-sustaining inflammatory configuration—an epigenetically encoded microglial phenotype that resembles trained immunity in peripheral macrophages and persists even after resolution of the initial insult (Huang et al., 2023; Neher and Cunningham, 2019; Zhang X. et al., 2022). Peripheral trained immunity has been proposed to contribute to sustained neuroimmune activation. Surgical or systemic inflammatory stimuli may induce epigenetic reprogramming of circulating monocytes, a phenomenon termed “trained immunity,” characterized by persistent pro-inflammatory responses including IL-1β, IL-6, and TNF-α release (Netea et al., 2020). Although direct clinical evidence remains limited, these systemic cytokines may modulate central immune homeostasis, potentially reinforcing microglial priming via soluble mediators rather than direct cell infiltration. This paradigm emphasizes targeting persistent immune cell states that underlie neuroimmune dysregulation, beyond merely suppressing acute inflammation.

## 3 A three-axis mechanism for inflammatory memory state

The persistence of microglial activation in POCD cannot be adequately explained by transient stimuli alone. Instead, it appears to originate from a feedback-coupled biological network—an interdependent triad of mitochondrial stress, chromatin imprinting, and phenotypic persistence that mutually reinforce one another (Zhang X. et al., 2022; Zhang L. et al., 2025). This “three-axis” model of inflammatory memory does not describe a sequence of independent processes but a co-evolving system that traps microglia in a pathologically sensitized state. Each axis contributes uniquely: mitochondrial stress acts as the initial signal integrator, epigenetic reprogramming stabilizes a maladaptive identity, and sustained polarization amplifies neurotoxic output. The following section focuses on the initiating role of mitochondrial stress.

### 3.1 Mitochondrial stress as the fast trigger

Among the earliest events after surgical insult is the emergence of mitochondrial dysfunction in central nervous system immune cells, particularly microglia. Surgery presents multiple metabolic challenges—hypoxia, anesthetic toxicity, and systemic inflammation—that converge upon mitochondria, leading to rapid alterations in microglial morphology and bioenergetics. Within hours post-injury, murine studies have reported swelling of mitochondrial cristae, elevated mtROS, and activation of redox-sensitive inflammatory signaling (He et al., 2022; Ye et al., 2016).

These stress responses are not incidental. mtROS and mitochondrial DNA (mtDNA), two potent damage-associated molecular patterns (DAMPs), act as immunogenic signals. mtROS facilitates lipid peroxidation and inflammasome activation via NLRP3, promoting caspase-1–dependent maturation of IL-1β and IL-18 (Antón and Traba, 2022; Han et al., 2021; Wu et al., 2019). Concurrently, mtDNA—hypomethylated and structurally akin to bacterial genomes—activates the cGAS–STING pathway once released into the cytosol (Yang et al., 2024). This induces type I interferons and NF-κB signaling cascades, contributing to an antiviral-like transcriptional profile within hippocampal microglia (Liu Y. et al., 2024). Notably, inhibition of either NLRP3 or STING alleviates postoperative cognitive deficits in rodent models, supporting a causal link (Yang et al., 2024; Zhang L. et al., 2025).

Microglial detection of these mitochondrial DAMPs is mediated through pattern recognition receptors such as NOD-like receptors (Zhang L. et al., 2025). But this detection is not a transient event; it leads to persistent transcriptional reprogramming involving IRF7 and chromatin-modifying complexes (Bowen et al., 2024; Saeki et al., 2024; Tanaka et al., 2015). These molecular signatures render microglia hyperresponsive to subsequent perturbations, a property known as trained immunity in peripheral innate cells (Neher and Cunningham, 2019; Zhang X. et al., 2022). Additionally, the impairment of mitophagy, particularly through PINK1–Parkin dysregulation, results in accumulation of dysfunctional mitochondria and continued DAMP release (Wu et al., 2021; Yao et al., 2024). This failure of mitochondrial quality control sets the stage for a feedback loop in which oxidative stress and immune activation sustain one another (Lv et al., 2024).

Cumulatively, the mitochondrial axis initiates and perpetuates inflammatory activation in microglia (van Horssen et al., 2019). Far from a transient trigger, mitochondrial dysfunction transforms microglia into long-term effectors through metabolic and redox instability (Sangineto et al., 2023; Wang et al., 2024). This process acts as the first pivot in the inflammatory memory circuit, preparing the cellular landscape for more durable epigenetic reprogramming.

### 3.2 Epigenetic reprogramming as the slow engraver

Where mitochondrial dysfunction primes the acute inflammatory reactivity of microglia, it is the epigenetic machinery that inscribes this state into a persistent transcriptional identity. Chromatin modifications, DNA methylation, and long non-coding RNA (lncRNA) coordination collectively reshape the regulatory landscape of microglia, transforming them into hypersensitive effectors of neuroinflammation (Cai et al., 2020; Yang X. et al., 2021). In this axis—the “slow engraver”—epigenetic alterations serve not as echoes of inflammation but as its lasting codex.

DNA methylation provides a parallel and complementary layer of transcriptional control. DNMT3a, a *de novo* DNA methyltransferase, is upregulated in neuroinflammatory and neurodegenerative conditions and targets promoter regions involved in mitochondrial metabolism and synaptic regulation (McGregor et al., 2025). These modifications repress oxidative phosphorylation genes and sustain glycolytic, pro-inflammatory phenotypes. Inhibiting DNMT3a reverses these changes, restoring metabolic flexibility and attenuating inflammatory output, indicating its role in locking microglia into a maladaptive state. LncRNAs add further complexity to this regulatory architecture. MEG3 and MALAT1, two well-studied lncRNAs, are upregulated in microglia under neuroinflammatory conditions (Cai et al., 2020; Li et al., 2020). MALAT1, similarly, binds EZH2 to mediate epigenetic silencing of anti-inflammatory regulators (Cai et al., 2020; Xu et al., 2022).

The net result is a transcriptionally poised but epigenetically locked microglial state. Single-cell chromatin accessibility profiling (scATAC-seq) demonstrates persistent rapidly induced upon re-challenge (Saeki et al., 2024; Zhang X. et al., 2022). This suggests that epigenetic reprogramming creates a landscape primed for pathological recall. Critically, these modifications are maintained independent of ongoing inflammatory stimuli, supporting the concept of microglial memory. Transcription factors such as PU.1, IRF8, and C/EBPβ orchestrate this process, linking early mitochondrial signals with durable chromatin remodeling (Saeki et al., 2024). Their activation patterns form regulatory loops that reinforce accessibility at inflammatory gene loci and silence neuroprotective programs (Unlu et al., 2007; Zhou et al., 2019). Thus, epigenetic reprogramming in POCD is not simply an outcome of prior activation—it is the mechanism that sustains it.

This second axis of inflammatory memory ensures that microglia retain a biased, hypersensitive state long after the inciting event has passed. As a result, even minimal perturbations can provoke exaggerated responses, sustaining neuronal injury and cognitive decline. In the context of POCD, this axis transforms inflammation from an event into a condition—a locked transcriptional program embedded in chromatin and reinforced by lncRNA scaffolds.

### 3.3 Microglial polarization as the executor and amplifier

With microglial inflammatory memory set in motion by mitochondrial danger signaling and epigenetic imprinting, the third axis—phenotypic polarization—emerges as the effector limb of persistent neuroinflammation. This axis is not a passive endpoint but an active amplifier that embeds and perpetuates the pathological program. At the center of this mechanism lies the sustained M1-like polarization phenotype: a pro-inflammatory state marked by upregulation of iNOS, CD86, and MHC-II and downregulation of markers such as CD206 and arginase-1 (Du et al., 2024; Zhao et al., 2019).

Under normal conditions, microglia exhibit plasticity between inflammatory and reparative states. In the postoperative brain, however, this flexibility is compromised. Prolonged M1 polarization has been observed for over two weeks in rodent POCD models, correlating with hippocampal synapse loss, impaired long-term potentiation, and memory deficits (Li et al., 2022; Qi et al., 2023). These M1-polarized microglia act as autonomous effectors, releasing TNF-α, IL-1β, and IFN-γ, which not only impair neurons directly but also fuel oxidative stress and mitochondrial dysfunction in glia and neurons alike (Qiu et al., 2016; Wang et al., 2015). Importantly, this phenotype feeds back into the mitochondrial and epigenetic axes (Ji et al., 2024). TNF-α has been shown to induce mitochondrial permeability and mtDNA release in neurons, reinforcing cGAS–STING activation in microglia (Liu Y. et al., 2024; Willemsen et al., 2021). Meanwhile, M1 signaling maintains NF-κB activation via sustained IκB kinase activity and transcriptional reinforcement at enhancer regions (Yang et al., 2024). Key transcription factors—including STAT1, and lncRNAs like MEG3—further consolidate this state by enhancing inflammatory gene expression (Ma et al., 2024; Przanowski et al., 2014). This phenotype is stabilized, in part, by suppression of anti-inflammatory programs involving STAT6 and PPARγ (Liu X. et al., 2024; Yao et al., 2023). Concurrently, the anti-inflammatory axis is suppressed. SIRT1, a regulator of mitochondrial homeostasis and NF-κB restraint, is downregulated after surgery, particularly in aged animals (Yan et al., 2019; Zhang J. et al., 2022). This loss disables metabolic resilience and accelerates polarization inertia. Microglia, in this context, cease to function as sentinels and instead become long-term amplifiers of neurotoxicity (Liu et al., 2025). Emerging single-cell transcriptomic studies reveal that microglia are far from homogeneous, instead comprising multiple phenotypic subsets beyond the classical M1/M2 dichotomy (Masuda et al., 2020). Among these, disease-associated microglia (DAM) represent a transcriptionally distinct subtype characterized by upregulation of TREM2 and APOE, expanding in contexts of chronic neuroinflammation and neurodegeneration (Keren-Shaul et al., 2017). These findings underscore that discrete microglial populations engage specific transcriptional programs depending on the nature and chronicity of the insult. Aging itself is known to increase microglial heterogeneity, as demonstrated by single-cell transcriptomic studies identifying pro-inflammatory and interferon-responsive clusters in aged murine brains, suggesting that the aged microglial landscape may predispose to exaggerated neuroimmune responses following surgical stress (Hammond et al., 2019).

**FIGURE 1:**
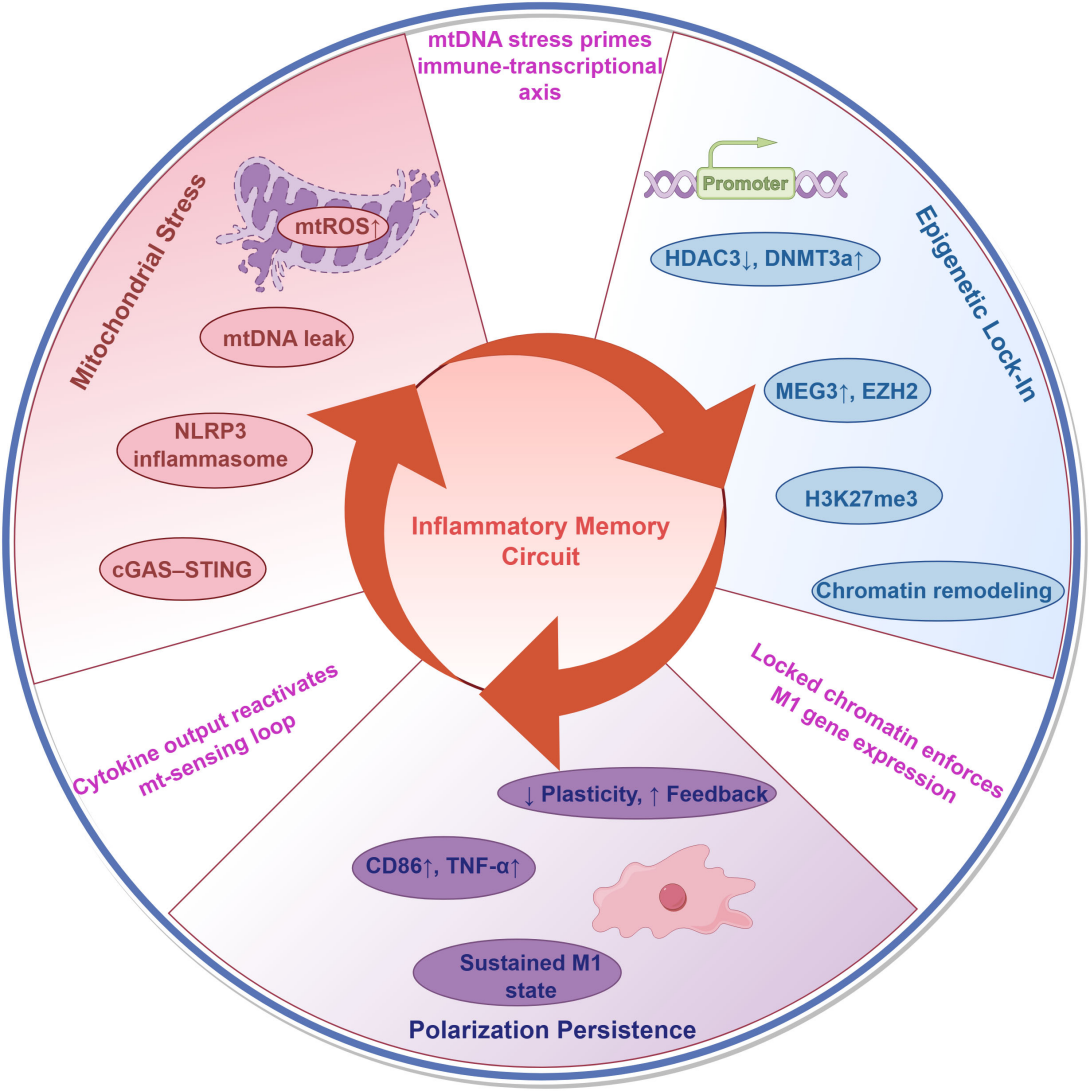
Inflammatory memory circuit in postoperative cognitive dysfunction (POCD). A closed-loop model linking mitochondrial stress, epigenetic lock-in, and sustained microglial polarization in postoperative cognitive dysfunction. Surgical stress induces mtROS and mtDNA release, which activate innate immune sensors (NLRP3 inflammasome and cGAS–STING). This response initiates chromatin remodeling via HDAC3 suppression and MEG3–EZH2–H3K27me3 axis engagement. The resulting epigenetic landscape enforces a fate-locked M1-like phenotype, characterized by TNF-α overproduction and loss of plasticity. These outputs further amplify upstream mitochondrial perturbation, completing a self-sustaining pathogenic loop.

This third axis thus closes the loop: what begins as a stress response becomes an encoded inflammatory phenotype, executed through persistent polarization and sustained by mitochondrial dysfunction and epigenetic reprogramming. The resulting system is no longer dependent on the initial insult—it has become its own driver of pathology. Collectively, these three axes—mitochondrial stress propagation, chromatin remodeling and epigenetic lock-in, and sustained polarization—do not operate in isolation. Instead, they reinforce one another in a self-perpetuating manner. To integrate these findings, we outline a unified mechanistic model: a circular “inflammatory memory circuit” that may underlie the persistence of postoperative cognitive dysfunction (Figure 1).

## 4 A closed-loop model of inflammatory memory in POCD

The persistence of cognitive dysfunction following transient surgical insult necessitates a shift from linear models of postoperative neuroinflammation to a more dynamic framework (Hovens et al., 2012; Liu et al., 2023). Rather than fading with the resolution of systemic signals, inflammation in POCD appears to be sustained by a recursive network of cellular reprogramming. We propose that this durability arises from a closed-loop “inflammatory memory circuit”—a self-reinforcing system in which mitochondrial dysfunction, chromatin remodeling, and phenotypic polarization are not merely parallel processes, but mutually catalytic nodes in a circular pathophysiological sequence. Once microglia cross a reactivity threshold, they enter a metastable state in which each axis of the circuit perpetuates the others, transforming a short-term perturbation into a long-term disorder.

### 4.1 Initiation: mitochondrial danger signaling

The loop is initiated by acute metabolic stress imposed by surgery and anesthesia, which elicits mitochondrial dysfunction in microglia (Yang et al., 2024). mtROS and mitochondrial DNA (mtDNA) are released as DAMPs, activating canonical innate immune pathways including the NLRP3 inflammasome and the cGAS–STING axis (Ji et al., 2024; Liu Y. et al., 2024). These pathways catalyze the production of IL-1β, IL-18, and type I interferons, establishing a rapid pro-inflammatory program that primes the immune transcriptome (Yang et al., 2024; Zhang L. et al., 2025).

### 4.2 Stabilization: epigenetic engraving of an inflammatory identity

Rather than resolving with the attenuation of DAMP signaling, this initial activation leads to chromatin reconfiguration. DNMT3a has been shown to enhance innate immune activation by upregulating HDAC9, which deacetylates TBK1 and facilitates downstream antiviral signaling (Li et al., 2016). The long non-coding RNA MEG3 interacts with chromatin remodelers such as EZH2 to modulate epigenetic repression of pro-inflammatory genes in postoperative cognitive dysfunction (Ma et al., 2024). This results in an “epigenetic lock-in,” rendering microglia hyper-responsive and transcriptionally biased even in the absence of external stimuli (Zhou X. et al., 2024).

### 4.3 Execution and amplification: polarization-driven feedback

These epigenetically biased microglia adopt a persistent M1-like polarization phenotype, marked by sustained NF-κB and STAT1 activity, high levels of TNF-α and IL-1β, and suppression of anti-inflammatory mediators like PPARγ and SIRT1 (Liu Y. et al., 2024; Yan et al., 2019; Zhang Q. et al., 2024). Crucially, the outputs of this phenotype feed directly into upstream axes: cytokines such as TNF-α exacerbate neuronal mitochondrial damage, triggering further mtDNA release and reinforcing cGAS–STING signaling (Ji et al., 2024; Yang et al., 2024). Thus, polarization functions as both the executor and amplifier of the circuit, transforming microglia into autonomous drivers of neuroinflammation (Kong et al., 2022).

### 4.4 Pathophysiological consequence: a self-reinforcing neuroimmune state

The convergence of these three axes creates a system in which the original insult is no longer required to maintain pathology. Mitochondrial dysfunction reinforces epigenetic priming; chromatin remodeling sustains polarization; and M1 polarization recycles mitochondrial stress. This circuitous architecture recasts POCD not as an extended response to injury, but as a state transition—one in which microglia have acquired a memory-like identity that persists beyond resolution and resists reversal. Aging also exacerbates key regulatory failures in microglia. In older brains, PINK1/Parkin-mediated mitophagy is impaired, leading to accumulation of damaged mitochondria that release mtDNA and trigger innate immune sensors (e.g., cGAS–STING and NLRP3), thereby locking microglia in a pro-inflammatory state (Gulen et al., 2023; Lv et al., 2024). Concurrently, aging is associated with pronounced epigenetic alterations in microglia that compromise their ability to regulate inflammatory gene expression. For example, several HDAC isoforms—including HDAC1 and HDAC3—are upregulated in senescent microglia and aged hippocampal tissue, correlating with enhanced microglial and senescence markers (Auzmendi-Iriarte et al., 2022). These epigenetic vulnerabilities likely render aged microglia more susceptible to adopting maladaptive, persistent activation states following surgical stress (Fonken et al., 2018).

By conceptualizing POCD as an emergent property of this inflammatory memory circuit, we provide a framework that explains both the delayed onset and the prolonged duration of symptoms. More importantly, this model identifies multiple nodal points—metabolic, epigenetic, and phenotypic—at which intervention may disrupt the loop and restore neuroimmune equilibrium.

## 5 Hypothesis testing and experimental strategies

To empirically validate the inflammatory memory circuit proposed in POCD, we outline a two-pronged strategy: temporal mapping of microglial state transitions, and targeted pharmacologic interventions aimed at disrupting key axes of the circuit. This approach is further supported by biomarker development to enable translational application and real-time clinical stratification.

### 5.1 Temporal mapping of state transitions

**FIGURE 2:**
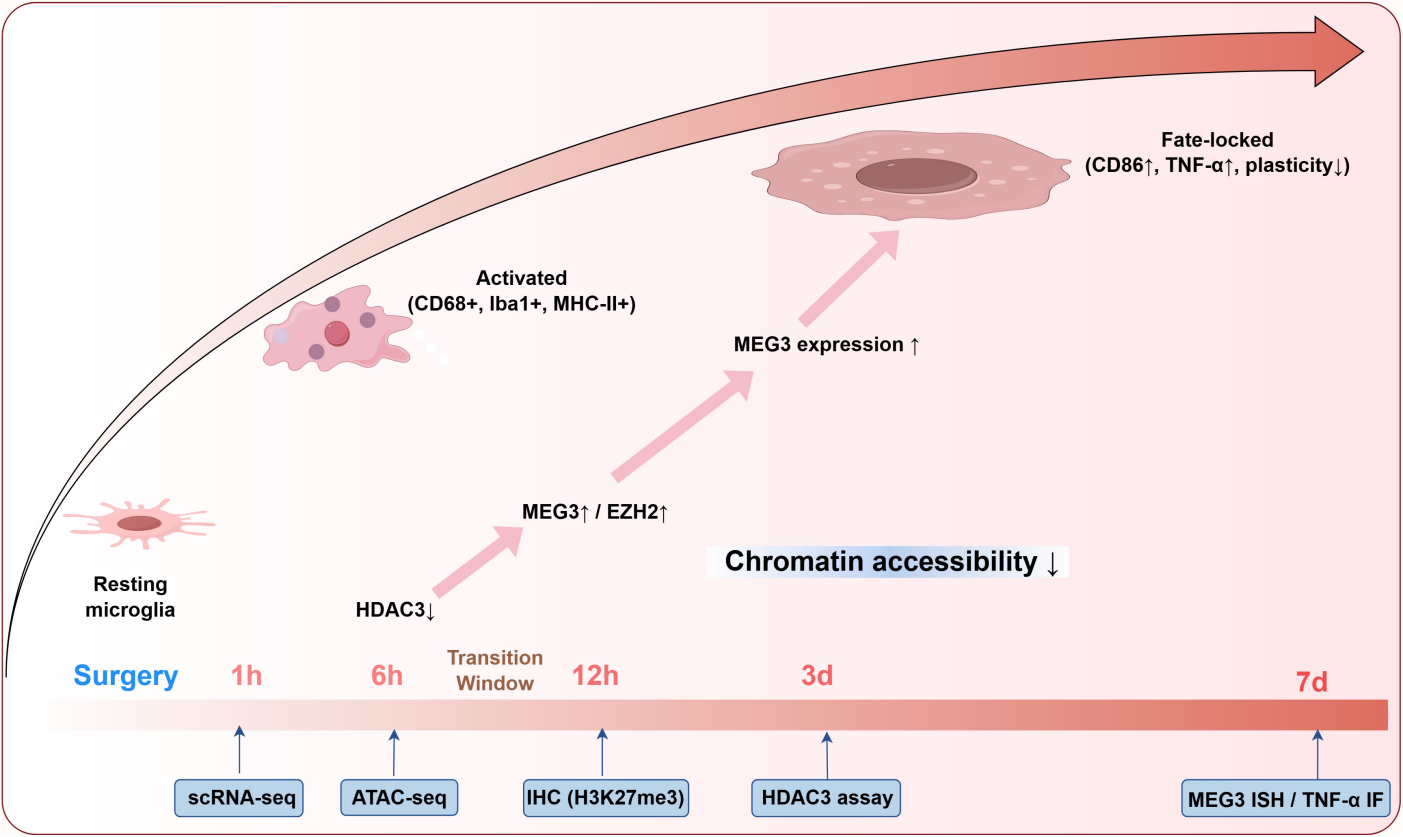
Microglial state trajectory and epigenetic transitions following surgery. This schematic summarizes postoperative microglial state transitions across defined timepoints (1 h–7 days), integrating phenotypic markers with regulatory mechanisms. The progression from resting to activated and fate-locked states corresponds with HDAC3 downregulation, MEG3/EZH2 upregulation, and increased H3K27me3 levels. Relevant detection strategies, including scRNA-seq, ATAC-seq, and immunohistochemistry, are aligned to each timepoint to support stage-specific analysis and potential therapeutic targeting.

A defining feature of the inflammatory memory model is the transition from transient activation to a metastable, memory-locked microglial state (Huang et al., 2023; Martins-Ferreira et al., 2021; Zhang X. et al., 2022). To resolve this trajectory, we propose longitudinal sampling at six critical postoperative timepoints—1, 6 h, 1, 3, 7, and 14 days—encompassing both the acute phase and the putative chromatin-locking window. These stage-specific changes are visualized in a trajectory schematic that links cellular states, molecular regulators, and detection strategies across the postoperative timeline (Figure 2).

In rodent models of postoperative cognitive dysfunction, microglia may be isolated from the hippocampus using fluorescence-activated cell sorting (FACS) based on canonical markers such as CD11b (Rayaprolu et al., 2020). Ingle-cell RNA sequencing (scRNA-seq) and ATAC-seq allow parallel profiling of transcriptome and chromatin accessibility in microglia. Focus should include mitochondrial regulators (e.g., TFAM, NDUFS3), polarization markers (iNOS, Arg1, CD86), and chromatin modifiers including HDAC3, DNMT3a, and EZH2. Accessibility changes at enhancer regions targeted by PU.1, IRF8, and NF-κB serve as epigenetic indicators of durable transcriptional reprogramming (Martins-Ferreira et al., 2021; Zhang X. et al., 2022). In parallel, mitochondrial stress should be quantified by cytosolic mtDNA abundance via qPCR and mtROS accumulation using MitoSOX fluorescence, which are validated indicators of mitochondrial damage in POCD-related microglial activation (Ji et al., 2024; Zhang L. et al., 2025). Immunohistochemistry should track co-localization of Iba1? microglia with MHC-II, cGAS–STING, and H3K27me3 to distinguish activation status and epigenetic locking, as evidenced in POCD and neurodegenerative models (Yang et al., 2024; Zhou X. et al., 2024). This multimodal approach enables differentiation of transient activation from irreversible microglial reprogramming and supports the hypothesis of bifurcation into an inflammatory memory state.

### 5.2 Pharmacologic interruption and biomarker development

A second axis of validation assesses whether early therapeutic intervention can interrupt or reverse microglial reprogramming. Here, agents targeting specific circuit components are administered at key timepoints before state-locking occurs.

For mitochondrial reprogramming, PGC-1α agonists such as ZLN005 may prevent mtDAMP release and subsequent inflammasome activation. In POCD models, ZLN005 administration significantly upregulated PGC-1α signaling, suppressed NLRP3 activation, and restored mitochondrial function (Wu et al., 2024; Zhang W. et al., 2024). Behavioral improvements in novel object recognition and fear conditioning tests were observed following treatment at early postoperative intervals (1–6 h), with parallel reductions in IL-1β, IL-6, and restoration of mitochondrial biogenesis markers (Wu et al., 2024). To simultaneously modulate mitochondrial and epigenetic axes, SIRT1 activators such as SRT1720 offer a dual mechanism: deacetylation of NF-κB and restoration of PGC-1α-driven mitochondrial biogenesis (Pang et al., 2024; Sung et al., 2024). These interventions are especially relevant in aged animals where SIRT1 expression is reduced and mitochondrial dysfunction prominent (Mitchell et al., 2014).

Age-stratified analyses are essential to define the temporal therapeutic window. In aged POCD models, epigenetic checkpoints such as Mef2C are rapidly lost, predisposing microglia to primed, inflammation-permissive states (Wu et al., 2023). If chromatin imprinting and loss of immune regulation occur within the first few hours after surgery, reversal strategies may become ineffective once microglia cross a transcriptional “point of no return” (Zhou X. et al., 2024). These findings support the model’s prediction of irreversible inflammatory memory transitions when early interventions are missed (Zhang X. et al., 2025). In parallel, the circuit model enables rational biomarker development.

Although direct evidence in POCD is still emerging, mitochondrial DNA (mtDNA) levels in blood have been shown to correlate with cognitive impairment in non-POCD human cohorts and may reflect systemic mitochondrial dysfunction (Tian et al., 2025). Plasma mtDNA has also been associated with neuroinflammatory phenotypes in geriatric populations and HIV-related cognitive decline (Johnston et al., 2022). Peripheral HDAC3 activity, as measured by fluorometric assays, has been implicated in epigenetic regulation relevant to cognitive decline, and its elevation is observed in both aged animals and systemic inflammatory models affecting cognition (Sathishkumar et al., 2016; Yang L. et al., 2022). Longitudinal tracking of these markers at 1, 3, and 7 days could identify patients at highest risk for POCD—even before behavioral symptoms manifest.

In aggregate, these strategies provide a mechanistic and translational pipeline: dissecting temporal dynamics, intervening pharmacologically, and predicting trajectory via liquid biopsy. The inflammatory memory circuit thus becomes not only a conceptual model, but an experimentally tractable and clinically actionable framework. Its success will hinge on identifying the precise point at which microglia shift from reversible responders to autonomous propagators of dysfunction—and on learning how to stop them before the circuit completes.

## 6 From inflammatory trigger to cellular state transition: a conceptual shift

### 6.1 Reframing POCD as a microglial state transition

The prevailing model of POCD frames it as a temporary response to acute insult—surgical trauma and anesthesia trigger peripheral inflammation, which transiently breaches the blood–brain barrier, activates glial cells, and disrupts neuronal communication (Qiu et al., 2023; Riedel et al., 2014; Terrando and Akassoglou, 2022). While conceptually intuitive, this linear framework fails to resolve a key paradox: how can a fleeting peripheral event produce cognitive decline that persists for weeks or months, especially in aged patients without prior cognitive impairment?

To reconcile this discrepancy, we propose a redefinition of the underlying pathology: POCD is not merely the downstream consequence of unresolved inflammation—it is the consequence of a fate transition within microglia. Following the initial insult, a subset of microglia fails to return to homeostasis and instead enters a metastable, maladaptive state. This transition is not defined by continued exposure to stimuli, but by an intrinsic transformation stabilized by mitochondrial dysfunction, chromatin remodeling, and sustained polarization (Suo et al., 2025; Ying et al., 2024). The change is not incremental but categorical—marked by a lowered activation threshold, rewired gene expression programs, and a loss of plasticity (Tan et al., 2023; Zhang X. et al., 2025). This concept reflects a broader biological principle. Microglia, like neurons, are highly responsive to contextual cues. Their identity is plastic, shaped by ongoing input from the tissue microenvironment, metabolic status, and epigenomic configuration (Cheray and Joseph, 2018; Lauro and Limatola, 2020). When these signals converge beyond a critical threshold—particularly through mtROS accumulation and cytosolic mtDNA release—they engage transcriptional circuits that rapidly shift toward inflammatory dominance (Lauro and Limatola, 2020; Nakanishi et al., 2011). Over hours to days, this response is consolidated: anti-inflammatory repressors are silenced, inflammatory enhancers become more accessible, and long non-coding RNAs such as MEG3 further entrench the pro-inflammatory identity (Tan et al., 2023; Ye et al., 2023).

What emerges is not prolonged inflammation, but the formation of a new cellular baseline. Microglia no longer fluctuate in response to external stimuli; instead, they adopt a fixed identity that continues to shape brain function long after the surgical insult resolves. Although direct evidence of microglial epigenetic memory in human POCD is currently lacking, clinical research has begun to explore translational biomarkers of neuroinflammation. For example, translocator protein (TSPO) PET imaging has been employed to detect central neuroinflammatory activity associated with postoperative cognitive decline in elderly patients (Forsberg et al., 2017). Additionally, microglia-associated biomarkers such as soluble TREM2 in cerebrospinal fluid and exosomal non-coding RNAs in plasma are under investigation as indicators of perioperative neuroimmune activation (Henjum et al., 2016). Future integration of single-cell transcriptomic and epigenomic tools in postmortem brain tissue may clarify whether surgery induces long-lasting microglial reprogramming in humans. POCD, from this perspective, is the clinical manifestation of glial reprogramming—a state transition in which immune sentinels become autonomous effectors of neurotoxicity.

### 6.2 Comparative paradigms in neuronal and glial reprogramming

The state-transition model proposed for POCD is not without precedent. Across multiple biological domains, transient perturbations have been shown to induce durable changes in cellular identity—a principle evident in neuronal plasticity, trained innate immunity, and astroglial fixation (Li et al., 2021; Muscat and Barrientos, 2021; Tercan et al., 2021).

In the nervous system, experience-dependent transcriptional plasticity is a cornerstone of memory formation. Neurons exposed to high-frequency stimuli undergo persistent alterations in chromatin structure at genes such as Arc, Egr1, and BDNF, modifying their responsiveness to future inputs (Ibarra et al., 2022; Penke et al., 2011). These transcriptional changes are stabilized by activity-dependent enhancers and histone modifications, facilitating memory encoding—but also contributing to pathologies such as epilepsy and chronic pain when dysregulated (Ding et al., 2017; Zhang X. et al., 2022). Similarly, microglia exposed to surgery-related stressors appear to “memorize” the insult, not through synaptic connectivity, but through stable shifts in inflammatory gene regulation (Neher and Cunningham, 2019; Wendeln et al., 2018).

The innate immune system provides a parallel in the concept of trained immunity. Contrary to long-standing dogma, monocytes, NK cells, and macrophages can develop memory-like properties after infection or inflammation (Netea et al., 2016; Netea et al., 2020). This training involves metabolic reprogramming (e.g., increased glycolysis) and epigenetic remodeling of inflammatory loci, producing a heightened response upon re-exposure to even unrelated antigens (Cheng et al., 2014; Saeed et al., 2014). Microglia in POCD appear to mirror this behavior: brief exposure to mitochondrial DAMPs generates a transcriptionally biased state, in which subsequent perturbations evoke exaggerated inflammatory responses (Schindler et al., 2018; Zhang L. et al., 2025). Astrocytes also exhibit a state-transition phenomenon in neurodegenerative diseases. In Alzheimer’s disease, for instance, astrocytes shift into the neurotoxic “A1” phenotype, characterized by synapse elimination, pro-inflammatory cytokine secretion, and persistent transcriptomic alteration (Hulshof et al., 2022). Importantly, this phenotype is not easily reversible and is maintained independent of ongoing external stimuli (Lopes et al., 2021). The parallels with M1-polarized microglia in POCD are striking: both glial cell types, once pushed past a functional tipping point, become active contributors to neural injury rather than protectors of homeostasis.

These examples underscore a unifying principle: cells across physiological systems can undergo stable, stimulus-independent reprogramming in response to transient perturbation. Whether in the form of memory consolidation, immune training, or gliosis, these transitions rely on an initial stressor interpreted through the lens of mitochondrial metabolism and epigenetic regulation (Liotti et al., 2022; Walters and Zovkic, 2015). The inflammatory memory circuit described here fits squarely within this framework, positioning POCD as one manifestation of a more general biological motif. By shifting focus from inflammation as a transient response to inflammation as a reprogrammed identity, this model realigns therapeutic objectives. It suggests that recovery from POCD may require more than anti-inflammatory suppression—it may necessitate interventions that reset or rewire the epigenetic and metabolic programs underlying microglial fate. To consolidate these mechanistic insights into actionable strategies, we compiled representative targets, candidate interventions, and measurable readouts across the inflammatory memory circuit. These entries reflect preclinical feasibility and potential translational applicability (Table 1).

**TABLE 1:** Therapeutic targets, intervention strategies, and biomarker readouts within the inflammatory memory circuit.

Mechanistic target	Intervention strategy	Biomarker readout	Detection method
HDAC3 Histone deacetylase 3	Selective HDAC3 inhibitor (e.g., RGFP966)	↓ H3K27me3 (repressive mark) ↑ chromatin accessibility	Immunohistochemistry (IHC) ATAC-seq
MEG3 Long non-coding RNA	Antisense oligonucleotide (ASO)	↓ MEG3 in serum exosomes ↓ EZH2 mRNA (target of MEG3)	cfRNA quantification qPCR/*in situ* hybridization
EZH2/H3K27me3 Epigenetic silencing module	EZH2 inhibitor (e.g., GSK126)	↓ H3K27me3 in microglia ↑ Synaptic plasticity genes (e.g., BDNF, PSD95)	IHC for H3K27me3 RNA-seq/qPCR for synaptic genes
Fate-locked microglia Persistent M1-like state	Immune reset: Minocycline or CSF1R inhibitor	↓ TNF-α expression ↑ microglial plasticity markers	Immunofluorescence (IF) Multiplex protein assays
Mitochondrial stress mtDNA release, ROS	Antioxidants or cGAS-STING inhibitors (exploratory)	↓ circulating mtDNA ↓ IFN-stimulated gene expression	Plasma cfDNA assay qPCR/ELISA

This table summarizes therapeutic targets within the inflammatory memory circuit, matched with intervention strategies and corresponding biomarkers. Detection methods span histone modification assays, cfRNA profiling, and immunofluorescence, providing a framework for both preclinical validation and potential clinical translation.

## 7 Translational implications: intervening in the state transition loop

Reframing POCD as a state-locked, microglia-mediated condition necessitates a corresponding shift in therapeutic approach. Traditional strategies that aim to suppress inflammation may prove inadequate once microglia have entered a maladaptive fate. If the persistence of POCD reflects not unresolved cytokine signaling but a durable reprogramming of immune identity, then interventions must act at or before the point of cellular transition. This perspective opens a new translational landscape: one centered on interrupting the trajectory toward pathological imprinting rather than extinguishing its aftermath.

### 7.1 Pharmacologic interruption of the inflammatory memory circuit

Three mechanistic targets emerge as promising candidates for therapeutic modulation: HDAC inhibition, lncRNA interference, and SIRT1 activation. Each strategy addresses a distinct axis within the inflammatory memory circuit and offers an entry point for preventing or reversing glial reprogramming.

Histone deacetylase inhibitors, particularly those selective for HDAC3, can restore acetylation balance at key regulatory loci and suppress the formation of transcriptional memory (Sun et al., 2022). Compounds such as RGFP966 and vorinostat have demonstrated efficacy in reversing microglial hyperreactivity and improving cognitive outcomes in preclinical neuroinflammation models (Zhang M. et al., 2020). In the context of POCD, their window of effectiveness is narrow. Administration within six hours of surgical insult is likely necessary to preserve epigenetic flexibility and prevent entrenchment of the inflammatory phenotype (Lin et al., 2024). lncRNA-targeted approaches offer a complementary axis of control. MEG3, shown to scaffold chromatin repressors and modulate splicing of inflammatory transcripts, represents an actionable node for antisense oligonucleotide (ASO) or CRISPRi-based suppression (Hasenson et al., 2022; Li et al., 2024). In rodent models, early inhibition of MEG3 dampens microglial reactivity and cytokine production (Li et al., 2020; Meng et al., 2021). Future delivery platforms such as CNS-penetrant exosomes or lipid nanoparticles could enable rapid, region-specific suppression of these transcriptional amplifiers in the perioperative setting. SIRT1 agonists, including SRT1720 and resveratrol derivatives, offer dual benefits: suppression of NF-κB transcriptional activity and restoration of mitochondrial biogenesis via PGC-1α signaling (Sung et al., 2024; Wan et al., 2016). In aged animals, where SIRT1 activity is intrinsically lower and mitochondrial resilience is impaired, such agents may prevent both the metabolic trigger and the epigenetic lock-in that underlies the inflammatory memory loop (Gano et al., 2014; Zeng et al., 2021). While bioavailability limitations remain a concern for some compounds, newer formulations are in development that may allow clinical application.

These therapeutics, if deployed early, hold potential to redirect microglial fate away from chronic reactivity. Success hinges not merely on their anti-inflammatory potency, but on their alignment with the timing of the state transition itself.

### 7.2 Molecular profiling for risk stratification

Given the narrow therapeutic window, early identification of high-risk individuals becomes critical. The inflammatory memory framework supports a multi-marker profiling strategy based on mitochondrial, epigenetic, and transcriptomic features.

Mitochondrial DNA released into circulation after tissue injury or surgery may act as a DAMP, promoting innate immune activation and systemic inflammation (Zhang et al., 2010). This can be quantified via qPCR in blood samples collected intra- or immediately postoperatively. Peripheral HDAC3 activity, measurable in blood mononuclear cells via fluorometric assays, may reflect central epigenetic shifts, especially when analyzed in tandem with systemic inflammatory profiles (Monisha et al., 2023; Sathishkumar et al., 2016). Serum exosomal MEG3 expression offers a glial-specific readout of transcriptional reprogramming and has been potentia in neurodegenerative and ischemic contexts (Li et al., 2020; Zhou T. et al., 2024). Its early upregulation could function as both a diagnostic biomarker and a pharmacodynamic target for lncRNA-directed interventions.

Combining these molecular indicators into a composite risk index, alongside clinical factors such as age, cognitive reserve, and surgical complexity, would enable stratification of patients into risk tiers. High-risk individuals could be prioritized for early intervention, improving both therapeutic efficiency and resource allocation.

### 7.3 The six-hours window: from insight to intervention

A consistent signal emerges across mitochondrial, epigenetic, and transcriptional domains: the transition toward a locked microglial state occurs rapidly—often within the first 6–12 h postoperatively (Ji et al., 2024; Zhang L. et al., 2025). This defines a narrow but actionable window during which microglial fate remains modifiable. Intervention after this point likely encounters cells that have already undergone chromatin remodeling and metabolic fixation. In such cases, treatment must shift from prevention to reversal—a far more complex and less reliable endeavor. Analogous to thrombolysis in stroke or steroid administration in spinal trauma, timing is everything. The success of anti-POCD strategies will depend not only on drug choice, but on the speed with which diagnosis and treatment can be initiated.

In sum, the closed-loop model of POCD not only redefines pathogenesis—it recalibrates intervention. Therapeutics targeting the early reprogramming phase, guided by dynamic molecular profiling, may offer a path to preserving cognitive function in the face of surgical insult. By breaking the feedback loop before it becomes self-sustaining, we shift the clinical paradigm from late-stage damage control to early-stage fate correction.

However, a strict “6 h window” for intervention is clinically impractical, so alternative neuroimmune-modulating strategies should be highlighted. One promising approach is transient microglial suppression and replacement via CSF1R inhibitors: by pharmacologically depleting resident microglia and allowing repopulation, one can reset the inflammatory state beyond the acute window (Zhang X. et al., 2025). Such microglial repopulation has been shown to attenuate postoperative neuroinflammation and cognitive deficits in aged models even when initiated outside of narrow time constraints. Dynamic Biomarker Monitoring: Similarly, delays in detecting peripheral biomarkers limit their clinical utility. To bridge this gap, intraoperative or real-time monitoring techniques could be employed – for example, rapid point-of-care assays or real-time CSF sampling during surgery – to track surges in neuroinflammatory markers and guide timely interventions. Central Validation of MEG3: Finally, while we propose lncRNA MEG3 in circulating exosomes as a microglia-specific biomarker, it lacks direct CNS validation. We should discuss correlating MEG3-containing exosomes with established neuroinflammation measures such as TSPO-targeted PET imaging or microglial markers in CSF to ensure this peripheral readout truly reflects central microglial activation. Linking peripheral MEG3 signals with TSPO-PET (which visualizes glial activation, albeit not microglia-exclusive) or CSF inflammatory profiles would strengthen its translational relevance.

## 8 Conclusion and future outlook

Postoperative cognitive dysfunction has long eluded mechanistic clarity, often attributed to transient neuroinflammation without a consistent explanatory model for its persistence (Muscat et al., 2021; Vacas et al., 2013). The inadequacy of acute-response paradigms becomes particularly evident in patients who exhibit cognitive decline long after systemic markers of inflammation have normalized (Peng et al., 2023). This review proposes a shift in perspective: POCD is not merely a delayed consequence of resolved inflammation but reflects a durable, maladaptive state transition in microglia—one that embeds the memory of injury into the brain’s immune architecture.

At the center of this framework lies a self-reinforcing “inflammatory memory circuit” composed of three tightly coupled biological axes. Mitochondrial stress initiates the cascade through the release of mtROS and mtDNA, triggering NLRP3 and cGAS-STING pathways. Epigenetic mechanisms—including dysregulated histone deacetylation, DNA methylation, and lncRNA-mediated repression—subsequently engrave a transcriptional identity biased toward hyperresponsiveness. Finally, sustained M1-like polarization operationalizes this memory into chronic effector activity, further damaging neuronal mitochondria and feeding back into the cycle. The result is a metastable microglial phenotype with lowered activation thresholds and impaired capacity for resolution—one that no longer requires ongoing inflammatory input to sustain its pathogenic state.

This model explains several otherwise puzzling clinical observations. It accounts for delayed-onset or non-resolving POCD despite apparent systemic recovery (Muscat et al., 2021). It clarifies the limited efficacy of late anti-inflammatory interventions, which fail to reverse fate-locked glial states (Terrando et al., 2010). And it predicts the heightened vulnerability of aging microglia, whose compromised mitophagy and epigenetic rigidity render them more susceptible to transition into a memory-locked phenotype (Ji et al., 2024; Zhang L. et al., 2025).

More importantly, the model establishes clear inflection points for therapeutic and diagnostic intervention. Because the inflammatory memory state is defined by a transition—rather than a gradient—its trajectory is theoretically measurable and interceptable. Markers such as circulating mtDNA, elevated HDAC3 activity, and serum MEG3 levels could provide early signals of fate specification, enabling pre-symptomatic risk stratification (Pinti et al., 2021; Yang X. et al., 2022; Ye et al., 2023). If administered during this early window—within approximately 6 h post-insult—agents such as HDAC inhibitors, SIRT1 agonists, or lncRNA-targeting oligonucleotides may prevent the closure of the pathological circuit altogether (Ma et al., 2024; Shi et al., 2020; Yang et al., 2020).

Future research must now move toward validating this trajectory-based model in clinical and translational settings. Longitudinal single-cell studies, real-time chromatin profiling, and non-invasive biomarker development will be key to identifying patients before cognitive symptoms emerge. Equally critical is the development of scalable, fast-acting therapeutic platforms that match the narrow timing requirements of intervention.

Ultimately, POCD should no longer be conceptualized as a lingering trace of surgical stress. It is a circuit-encoded disorder of microglial fate, in which temporary perturbation becomes enduring dysfunction. Yet this model also opens space for therapeutic optimism: because state transitions are dynamic, they are not necessarily irreversible. If caught early enough, the trajectory toward cognitive decline may be diverted—restoring the brain’s innate immune balance and preserving cognitive integrity after surgery.
